# Older people’s adherence to community-based group exercise programmes: a multiple-case study

**DOI:** 10.1186/s12889-017-4049-6

**Published:** 2017-01-25

**Authors:** Clare Killingback, Fotini Tsofliou, Carol Clark

**Affiliations:** 1 Royal London House, R601, Faculty of Health and Social Sciences, Bournemouth University, Christchurch Road, Bournemouth, BH1 3LT UK; 2 Royal London House, R315, Faculty of Health and Social Sciences, Bournemouth University, Christchurch Road, Bournemouth, BH1 3LT UK; 3 Royal London House, R603, Faculty of Health and Social Sciences, Bournemouth University, Christchurch Road, Bournemouth, BH1 3LT UK

**Keywords:** Adherence, Physical activity, Community-Based exercise programme, Older people, Multiple-Case study

## Abstract

**Background:**

Physical inactivity is a global phenomenon, with estimates of one in four adults not being active enough to achieve health benefits, thus heightening the risk of developing non-communicable diseases. In order to realise the health and wellbeing gains associated with physical activity the behaviour must be sustained. Community-based group exercise programmes (CBGEP) utilising social supports have been shown to be one means of not only increasing activity levels for older people, but sustaining physical activity.

A gap in the literature was identified around older people’s long-term adherence to real-life CBGEP within a UK context. This study therefore sought to address this gap by understanding older people’s ongoing adherence to CBGEP with a view to gaining further insight about which factors contribute to enabling people to sustain their physical activity levels.

**Methods:**

A multiple case study research design was employed to understand older people’s (≥60 years, *n =* 27) adherence (≥ 69%, for ≥ 1 year) to three current CBGEP in the South- West of England. Qualitative data (participant observation, focus groups, documents, and interviews) were collected and analysed using inductive thematic analysis followed by the analytic technique of explanation building. Quantitative data were analysed using descriptive statistics and used to set the context of the study.

**Results:**

The current study offers five unique insights into real-life programmes which have been successful in helping older people maintain adherence for a year or longer. These included: *factors relating to the individual*, the *instructor* (particularly their personality, professionalism and humanised approach), *programme design* (including location, affordability, the use of music, and adaptable exercise content), *social features* which supported a sense of belonging, and *participant perceived benefits* (physical and psycho-social). These all served to explain older people’s adherence to CBGEP.

**Conclusions:**

These factors related to participant adherence of CBGEP must be considered if we wish to support older people in sustaining a physically active lifestyle as they age. These findings are of interest to practitioners and policy makers in how CBGEP serve to aid older people in maintaining a physically active lifestyle with a view to preventing non-communicable diseases and in maintaining social connectivity.

## Background

The public health burden of physical inactivity in the UK is substantial with an estimated cost to the National Health Service (NHS) of approximately £1.06 billion per year (National Institute for Health and Care Excellence) [1]. As such, physical inactivity has been said to be “one of the most important public health problems of the 21^st^ century” [2] (p 1).

The role of sustained participation in physical activity (PA) throughout the life-course provides some of the best prospects for ageing well [3]. Regular PA has been shown to reduce the risk of chronic diseases such as ischaemic heart disease, hypertension, type 2 diabetes, osteoporosis, some cancers, and depression [4]. Maintaining a PA lifestyle has also been shown to reduce the risk of all-cause mortality [5, 6]. A growing body of evidence is developing regarding the cognitive benefits of sustained PA in older people as well as improved quality of sleep, health-related quality of life, and lower risk of falls [2, 4].

The importance of being physically active through life and exercising with others is well acknowledged but approximately half of participants who commence an exercise programme will drop out within the first six months [7, 8]. Reviews of community-based group exercise interventions for older people have shown more favourable adherence outcomes with adherence rates of between 69.1–75% [9, 10].

Not only have community-based group exercise programmes (CBGEP) been shown to increase PA levels for older people, they have been found to provide functional improvements such as increased mobility, flexibility, and upper and lower limb function [11–18]. CBGEP also have positive effects on participants’ subjective sense of wellbeing, balance and fear of falling, and health related quality of life [18, 19].

One key facilitator for influencing older people’s adherence to CBGEP is the role of social support and its ensuing networks [9, 20, 21]. This is important because the quality and quantity of a person’s social relationships are a key health determinant, not only in terms of mental health but also in relation to morbidity and mortality [22]. Individuals with adequate social relationships have been found to have a 50% greater likelihood of survival when compared to those with limited social support [23]. The extent of this effect was argued to be comparable with cessation of smoking [23]. It has been suggested that there is a need to explore types of intervention that offer older people the opportunity to interact with others whilst increasing PA levels within real-life programmes within a community settings [9, 24].

The current study offers a unique insight into real-life programmes (i.e. not under laboratory conditions or research trial conditions) which have been successful in helping participants maintain adherence for a year or more. Specifically, the aim of this study was to understand how and why older people (≥ 60 years) have sustained long-term adherence (≥ 69.1% for ≥ 1 year) to three CBGEP in the South West of England. Ethical approval for the study was obtained from Bournemouth University Research Ethics Committee (reference number 5103). All participants gave written informed consent.

## Methods

### Study background and design

Case study research seeks to investigate contemporary phenomenon using a multi-faceted approach by relying on multiple sources of evidence [25]. Case study research is strong on reality, allowing the researcher to focus on a ‘case’ whilst retaining a holistic and real-world or real-life perspective [25–27]. A ‘real-life’ perspective implies studying programmes which are not carried out under trail conditions [28]. A case study approach permitted an in-depth investigation with the use of multiple sources of evidence (questionnaires, archival records, participant observation, focus groups, interviews and documentation) thus allowing the cases to be understood in a multifaceted way.

An evidence database was utilised in order to store the considerable amount of data collected in an organised and accessible manner [25]. The use of a case study database also helped in dealing with issues with regards to validity and reliability as the data could easily be retrieved [25]. This served to maintain a chain of evidence such that assertions presented during the analysis could be tracked back to their original evidence source [25].

### Sampling strategy

The cases in the current study were three purposively sampled CBGEP. These were selected to extend understanding of programmes which have been ‘successful’ in helping participants adhere to CBGEP for ≥ 1 year. This was selected based on NICE Behavioural Change guidelines [29] which recommend regular attendance of a year or more to bring about a long-term change in behaviour. Successful cases were also selected based on their programme retention rate, i.e. proportion of participants who are still attending the programme for ≥ 1 year in the year prior to data collection. To be included in the study CBGEP were required to have a programme retention rate of ≥ 69.1% as selected based on a recent quantitative synthesis of CBGEP adherence for older people [9]. Cases were further selected to reflect a range of socio-economic backgrounds based on total community level deprivation score [30]. Three cases were chosen to allow comparisons and understand whether results were consistently replicable in order to aid a more robust explanation of theory/propositions [31].

### Data collection

#### Questionnaires and archival records

Questionnaires were used to collect data to assist in understanding the demographic, individual lifestyle features (physical activity, diet, alcohol consumption and smoking) [32, 33] and socio-environmental characteristics [34, 35] of older people who adhere long-term to community-based group exercise programmes.

Archival records in the form of participant attendance records were utilised to calculate the programme and individual participant adherence rates. The programme adherence rate was used to reflect the overall retention rate of the programme. This was calculated as the percentage of participants who had adhered to the programme for ≥ 1 year in the year prior to data collection. The individual participant adherence rate was the percentage of sessions attended by each participant. This was calculated as the proportion of attended sessions relative to offered sessions and expressed as an adherence percentage [36].

#### Participant observation

Participant observation was chosen to allow for unstructured observations, providing a deeper understanding of context which would only be possible through personal experience [37]. The first author (CK – a Caucasian female in her late 30s) attended each CBGEP for six – eight weeks. Detailed handwritten notes were made immediately after the end of each session of participant observation. These handwritten notes were then typed and expanded in a Microsoft Word document at the earliest available opportunity and stored on the case study database.

#### Focus groups

Focus groups were chosen since they are known to explicitly generate data via the process of group interaction [38]. They also provided the opportunity for individuals to build on the answers of others in the group and in doing so generate new ideas [39]. The interaction with others, in agreement or disagreement allowed participants to be exposed to a broader range of opinions. Furthermore, the group dynamic allowed more time for the participants to reflect on their opinion [40].

All focus groups were conducted at the location of the CBGEP for participant convenience. They were digitally recorded following informed consent. Recordings were transcribed verbatim by the researcher using NVivo 10 [41]. Any identifiers were removed at transcription and individual participant numbers were assigned to their responses to maintain anonymity.

#### Documentation

Documents per se may not necessarily answer the research question but are seen as being an important part of the evidence base [42]. They are useful in verifying evidence from other sources and thus should be explicitly included as part of the data collection plan [25]. Documentation for this study was sourced in two ways. The first strategy of document retrieval involved using Internet searches for evidence related to the CBGEP. Secondly, the programme instructors were asked to provide documents relating to the programme, including, the structure, funding, implementation, goals, and philosophy. All documents were stored as part of the case study database in either electronic or paper format.

#### Semi-structured interviews with programme instructors

Interviews were digitally recorded with the individual instructors following informed consent and followed a semi-structured interview guide. Recordings were transcribed verbatim by the researcher using NVivo 10 [41]. Identifiers were removed at transcription to maintain anonymity.

### Data analysis

The analytic strategy employed for the qualitative data (participant observation, focus groups, interviews, and documentation) was inductive thematic analysis [43]. Authors FT and CC independently cross checked sections of qualitative data analysis by comparing the codes and themes to the transcripts and text. This assisted the interpretation, definition, and refinement of themes [44].

To assess the questionnaire data (lifestyle, social and socioeconomic data) descriptive statistics were performed. Since the data were non-parametric the median and interquartile range (IQR) was used to assess the central tendency and measure of spread [45]. These data were used to add further to the contextual understanding of the participants. Archival records were used to understand the participant adherence rates of each case and were reported descriptively.

#### Theoretical propositions

Yin [25] argues for the distinct inclusion of a theoretical framework or propositions to guide data collection and analysis. The case, after all is “an opportunity to shed empirical light about some theoretical concepts or principles” [25] (p 40). This study drew on the results of a qualitative systematic review in order to formulate theoretical study propositions [9]. This led to six theoretical study propositions which were considered as being noteworthy in influencing participant adherence to CBGEP: individual behaviour, instructor, programme design, social connectedness, participant perceived benefits, and energising and empowering effects.

#### Explanation building

The analytic technique of explanation building was employed to aggregate the qualitative findings across the three cases with the goal of seeking to build an explanation of the role each proposition played in participant adherence to CBGEP. Explanation building occurred in a narrative, iterative format. The findings from each case were compared to each theoretical proposition and the propositions were then revised based on the empirical evidence [25].

## Results

### Case descriptions

A summary of participant demographic, socio-environmental features and adherence by each case can be seen in Table 1.

**Table 1:** Participant demographic, socio-environmental features and adherence by case

Variables	Case 1 *n =* 14	Case 2 *n =* 5	Case 3 *n =* 8
Age, years, Median (IQR)	70.5 (10)	70 (5.5)	68.5 (4.5)
Gender, n (%)			
Male	3 (21)	1 (20)	1 (12.5)
Female	11 (79)	4 (80)	7 (77.5)
BMI, n (%)			
Normal	10 (71.4)	0	4 (50.0)
Overweight	3 (21.4)	3 (60)	3 (37.5)
Obese	1 (7.2)	2 (40)	1 (12.5)
Current relationship status, n (%)			
Married	10 (71.4)	4 (80)	5 (62.5)
Divorced	1 (7.2)	0	1 (12.5)
Widowed	3 (21.4)	1 (20)	1 (12.5)
Other: Lives with partner	0	0	1 (12.5)
Neighbourhood socio-economic status (deprivation rank), Median (IQR)	29147 (11226)	26291 (10865.5)	11859 (7524)
Highest education level, n (%)			
Some secondary school	2 (14)	0	0
Completed secondary school	6 (43)	3 (60)	3 (37.5)
Trade / technical / vocational	6 (43)	2 (40)	3 (37.5)
University	0	0	2 (25)
Current Employment, n (%)			
Full time	0	0	0
Part time	1 (7)	0	0
Retired	13 (93)	5 (100)	8 (100)
Programme adherence rate	69.4%	71.0%	77.8%
Individual participant adherence rate, Median (IQR)	74% (16.0)	91% (19.5).	73.5% (39.0).

#### Case 1

Case 1 was a privately run, non-profit CBGEP for older people, and individuals with chronic health conditions. The CBGEP is held in a town in the South West of England with a population of approximately 7,000 people [46]. The exercise programmes were, multicomponent programmes involving aerobic, strengthening, balance, and flexibility exercises lasting an hour and aimed at improving older peoples overall functional ability, health and wellbeing.

#### Case 2

Case 2 was a green exercise referral scheme whereby NHS health care practitioners referred patients for an outdoor exercise programme. The CBGEP took place outdoors at a country park in the South West of England. The exercise programme lasts for approximately one hour. The routine consisted of a warm up period of walking and gentle aerobic exercises followed by walking interspersed with strength, coordination, balance exercises, and stretches.

#### Case 3

Case 3 was a privately run, non-profit CBGEP for older people. This CBGEP was held in a seaside town in the South West England with a population of approximately 65,000 [46]. The exercise programme was multicomponent consisting of aerobic exercises, circuits (alternating cardiovascular and resisted exercises), strengthening, balance, coordination exercises, and stretches and lasted for one hour.

### Study propositions

Theoretical propositions as derived from the literature were utilised in order to understand how and why participants had sustained ongoing adherence to CBGEP [9]. The results are presented in light of each proposition.

#### Proposition one: factors related to the individual and CBGEP adherence

This proposition was concerned with factors related to the individual and the role this played in influencing participant adherence to CBGEP. Based on the findings from the three cases in this current study, the following aspects related to the individual were explicitly evident: preferences for a non-gym environment, current circumstances, personal motivators, and the role PA had played across participants’ lives.

Many individuals commented that they lacked the discipline to engage in exercises at home whilst gyms were seen as boring or isolating. Instead, participants preferred being together and exercising as a group. Additionally, there was a personal preference of appreciating the routine of the CBGEP and the structure this added to their week.

Some exercises were worked into the daily lives of participants such as neck or back exercises at home, or leg exercises whilst waiting for the bus. However, doing more formalised exercises at home did seem harder for individuals to achieve. The regular timing and routine of the CBGEP helped bring a discipline since it became part of the cycle of their week.

Participants commented on the various motivators which supported them in continuing to exercise. Participants expressed a desire to lose weight, stay active or to keep independent and healthy as they aged. In many ways maintaining independence was seen as vital because *“that’s the whole game”* (Female, 72 years, Case 3, FG - Focus group). There seemed to be a fear of losing independence, especially for those who lived alone and was thus a key motivator. Participants recognised that the more active they were, the longer they would be able to maintain their independence in daily life.

*"And my husband said one day, well why are you doing all this exercise? And I said to keep me healthy into my old age which hopefully I've done. You know. When you can't do things as well now as you did 2 or 3 years ago but you're still making the effort."* (Female, 81 years, Case 1, FG)

For participants who acted in the role of main carers for their partners, the CBGEP was seen as *“my little bit of me time”* (Field notes, Case 3) when they did not have to function in their caring role. Those who were carers also understood the importance of remaining fit and well so they could continue in those caring roles. Participants who lived alone commented on the simple fact that the CBGEP served as a reason for them to leave their homes which was an important motivator.

The social versus exercise drivers were noted to serve as different motivations for individuals. For example:

*“I think to be fair some people do exercise more than they chat and others chat more than they exercise. But then you're going to get that in a group aren't you, because everybody's there for different reasons, aren't they.”* (Female, 77 years, Case 3, FG)

Participants expressed a range of physical activity levels across their lifespan. This varied from those who had regularly engaged with exercise in aerobics classes, gyms, line dancing or bike riding to the more inactive:

*“I've never exercised in my whole life, apart from being at school when I used to do boxing, but since leaving school I've never exercised, never.”* (Male, 67 years, Case 2, FG)

At an early age, having schools that encouraged activity and provided opportunities to engage in sports seemed to help activity become a part of participants’ lives. For those who had exercised throughout their lives they noted that the mode of exercise had changed as they aged. For example, they could no longer ride a bike since their balance had deteriorated and managing their own garden was now too physically demanding.

The findings from this study would suggest that factors relating to the individual such as aspects of a participants personality, current circumstances, preferences for a non-gym environment, personal motivators and history of physical activity engagement are noteworthy in influencing participant adherence to CBGEP.

#### Proposition two: instructor and CBGEP adherence

This proposition is related to the role of the exercise instructor in influencing participant adherence to CBGEP. There was evidence from all three cases that participants found the instructor to be key in aiding their ongoing adherence. Specifically, the features of the instructor in relation to participant adherence included the instructors’ personality, their professionalism and their humanised approach.

The importance of the instructor’s personality was noted in a variety of ways. For example, the instructors were seen to demonstrate a care and concern towards individual participants. *“*[Instructor’s name] *really cares for her people, which is a great thing. She worries about people.”* (Male, 67 years, Case 2, FG)

The instructors were described as *“the leading light”* (Female, 68 years, Case 1, FG) or *“the lynchpin”* (Female, 72 years, Case 3, FG). The instructors had built a level of rapport with participants such that they provided a feeling of being at home within the group.

The instructors brought a sense of fun to the groups. Particularly during some of the choreographed warm up or coordination exercises:

*“The instructor added more coordination exercises with warm up today which made it a bit harder (left, right, then double arm). This made lots of people laugh because they couldn’t do it. She [the instructor] said if it’s too hard don’t worry but as a participant observer it was really fun to be a part of and watch the banter back and forth.”* (Field notes, Case 3)

The instructors were described using words such as enthusiastic, inspirational, jolly, lively, and encouraging.

*“If you haven't got a rapport with your instructor you just don't come back. We all feel quite at home here.”* (Female, 68 years, Case 1, FG)

The professionalism of the instructors were evidenced in them being aware of individual participant health conditions and in the ensuing, discrete advice offered to participants about working within their personal physical boundaries. The instructors did not judge or criticise participants in relation to their exercise ability. Instead, the instructors recognised individual participant physical limitations and adapted exercises accordingly. This personalised approach was highly valued and helped encourage participants to work to their own abilities.

*“*[Instructor’s name] *is a brilliant instructor and she can tell if you're not feeling quite at your best.”* (Female, 68 years, Case 3, FG)

The instructors commented that felt they were friendly, caring people who sought to be open and approachable:

*“I always hope that I'm quite an open person and I'm quite approachable because I think it's really important with the referral that you're somebody that people can open up to and talk to. That you're an approachable, friendly person that cares.”* (Instructor, Case 2)

The instructors prioritised participant safety. This was noted in regular reminders for participants to work within their pain free limits and in exercise adaptations for the varied levels of ability. The instructors also explicitly sought feedback from participants to ensure they were managing and benefitting from any changes in the exercise regime without undue strain.

The instructors treated participants in a humanised way. This was noted through participant’s awareness of the instructors’ level of care and responsibility for them. This in turn acted as a motivator for them to keep coming:

*“I think I'd feel I'd let her down as well if I didn't come. Because she puts all her efforts into keeping us fit and healthy and I'd think no, she's doing her best.”* (Female, 68 years, Case 1, FG)

The instructors also brought a sense of fun to the group in through their relaxed, informal manner that seemed to make exercise more enjoyable. Whilst it was understood that the instructors were not necessarily the predominant reason participants adhered, it was recognised that their leadership did contribute.

The instructor’s personality, professionalism and humanising approach was noteworthy in influencing participants’ ongoing adherence to community-based group exercise programmes.

#### Proposition three: programme design and CBGEP adherence

This proposition is related to the way the programme was designed, structured, and delivered. It includes features of time, location, cost, and structure including safe, adaptable exercise content.

Participants viewed the cost as being reasonable and *“a small price to pay”* (Female, 72 years, Case 2, FG). However, participants would not have wanted the cost to be any higher. The locations of the programmes were noted to be helpful inasmuch as they were local. As such, participants did not have to travel far from their home and could easily come by car. Being local also afforded participants the opportunity to meet other people within the area thus expanding their network of friends: *“I probably know more people now through doing these walks, through doing the exercises”* (Female, 73 years, Case 2, FG).

Participants in the indoor CBGEP expressed a preference for the use of music as they exercised which was seen as a motivator and as a means of entertainment (Case 1 and Case 3).

*“Yeah I think it's a good motivator…I think music does help. I find it. You know I'll be saying, where's the Christmas CD? Oh yeah, come on, let's have a bit of entertainment along with it. You know, it all helps.”* (Male, 74 years, Case 1, FG)

The structure of the CBGEP appeared to be helpful in participants continued engagement. The professional way that the CBGEP was run with exercises to a high standard of safety was important: *“she's not going to be asking us to do things that aren't good for us”* (Male, 68 years, Case 1, FG). Exercises were readily adapted to the individual depending upon their ability. Thus, the individual nature of the exercises was appreciated so that participants could work at their own level without pressure or judgement as well as the fact that it exercised the whole body rather than just focusing on balance or muscle strength.

#### Proposition four: social aspects and CBGEP adherence

This proposition related to the social aspects of the CBGEP describing the sociable, friendly, group aspects of the programme that participants noted to be important in relation to adherence to the exercise programme.

Participants appreciated the sociability of the exercise programme.

*"But it's the social aspect is good really because it makes you communicate and you know forget your own, forget yourself."* (Female, 77 years, Case 1, FG)

The social features of the programme were particularly important for those who lived alone.

*"I mean I've had somebody staying with me for several weeks and you know to go back last Monday and the house was quiet you know, it's different when you've got somebody at home…but yes it definitely makes a difference if you're on your own." (*Female, 78 years, Case 1, FG)

These social aspects aided adherence: *“I think it’s because it’s sociable, we like coming”* (Female, 67 years, Case 2, FG). Although it was noted that it took time to get to know people and build friendships but as the months went on they felt more at ease with one another. Participants viewed each other as nice people, which led to the development of new friendships which was seen as a meaningful benefit of attending.

*“That's a big thing I think, getting to know different people. Not people who are just your neighbours sort of thing but people further afield.”* (Female, 72 years, Case 2, FG)

Sharing advice appeared to be an overflow of the social nature of the group. This was evidenced in the form of health tips, beneficial exercises, and encouragement with doing exercises. Having shared experiences of similar health problems, medications, or comparable family challenges meant that participants felt they were *“in the same boat”* (Female, 78 years, Case 1, FG). The support aspect was marked in them finding a place to share frustrations or enquiring after one another’s health.

There was an awareness of the varying levels of ability between participants due to their different health conditions. This appeared to add to a sense of belonging since none of them were *“the odd one out”* (Female, 67 years, Case 2, FG). This helped them understand their own health challenges in the context of others.

*“And because people have got different things wrong with them, you know, you appreciate, you think well I'm quite lucky.”* (Female, 67 years, Case 2, FG)

Participants interacted with a level of care and concern for one another. For example, participants made sure everyone had a drink at the water break and were inclusive in conversations. This concern for one another extended beyond the boundaries of the CBGEP. For example, in Case 1, on one occasion, a participant was unable to attend for several weeks due to a fall and subsequent wrist fracture. When the instructor informed the group of this situation a participant requested her address so she could visit. There were elements of altruism in how they supported each other:

*“It makes you think of other people as well. I think because it's like* [participant’s name] *has not been coming because his wife is ill. You know we've all heard that his wife is ill so I feel that if he comes I'll have a chat with him and see how things are going to encourage…But if you think about somebody else it gives you a warm feeling I think.”* (Male, 74 years, Case 1, FG)

The group dynamic appeared to be linked to the highly social environment. Participants appreciated exercising as a group as opposed to their perceived impression of the more lone nature of individual gym programmes when *“nobody else talks to you”* (Female, 67 years, Case 2, FG) or were viewed as *“intimidating”* (Female, 68 years, Case 3, FG) and from a physical point of view the equipment was difficult to manage due to health limitations. The fact that they were not discouraged from socialising at various points in the classes added to the positive, entertaining group dynamic.

Participants felt that exercising in a group was easier than exercising alone at home. This was partly due to being away from the distractions of home, establishing an exercise routine, and exerting themselves more when exercising in a group. The notion of being together as a group also helped create a positive exercise environment:

*“…I think the extra bit is being with a whole group of other people who are similarly being aided in some shape or form. It's a sort of group feel as well. Being together, yes, I think this is a different kind of feel better because you're in a group together enjoying it.”* (Female, 72 years, Case 3, FG)

#### Proposition five: participant perceived benefits and CBGEP adherence

This proposition refers to the perceived physical and psycho-social benefits which participants reported as being important outcomes in contributing to adherence. The exercises helped participants maintain their health, manage their chronic health conditions (such as neck or back problems), and prevent health problems (such as incontinence).

*"…the other one I do daily…the pelvic floor because I noticed signs of problems and they've gone."* (Female, 77 years, Case 1, FG)

The physical gains of weight loss, improved balance, cardiovascular fitness, strength, increased walking ability, coordination, improved sleep and more energy were important helping maintain attendance. The CBGEP also acted as a mood lifter.

*“…in the first 6 months I lost over a stone in weight and I got a lot healthier. In fact, I was keeping up with everybody in the front. From being at the back of the group I became at the front of the group.”* (Male, 67 years, Case 2, FG)

Participants also expressed broader perceived benefits. For example, the social benefits were noted in that *“It* [the exercise programme] *gets you out of the house”* (Female, 78 years, Case 1, FG). There was a sense of enjoyment and feeling good about themselves; feeling cheerful, happier, encouraged, improved wellbeing, and a degree of achievement. Participants noted a sense of satisfaction and enjoyment from being involved in the exercise programmes. They had the view that *“…exercise should be enjoyable”* (Female, 63 years, Case 1, FG).

*“But I think it must be enjoyable because we wouldn't come if it wasn't would we? We wouldn't come just to keep fit would we really?…And we do pay therefore you're not going to pay to be unhappy.”* (Male, 71 years, Case 3, FG)

#### Proposition six: energising and empowering effect and CBGEP adherence

This proposition is related to the role in which energising or empowering effects may have in influencing sustained participant adherence. As a key influencing proposition there is limited evidence in this study to support the role of energising and empowering effects in being noteworthy in supporting sustained participant adherence.

Participants from Case 1 commented that they had more energy after attending the programme. Others commented on the positive, energising environment which was created when they all exercised together. Some participants in Case 3 found that attendance at the programme led to an energising effect as they felt invigorated after the CBGEP.

An empowering effect was implied by participants in Case 2 as they commented on the sense of achievement they felt after having completed the programme.

## Discussion

This multiple-case study was conducted to understand how and why older people (≥ 60 years) have sustained adherence (≥ 1 year) to real-life CBGEP. The analytical technique of explanation building was employed to seek to build an explanation of the role of six theoretical propositions as derived from the literature might influence participant adherence. Five of these propositions were found to be noteworthy in influencing ongoing adherence. The sixth proposition (related to the energising and empowering effects) was discounted due to a lack of cumulative evidence following the explanation building technique.

Firstly, the importance of *factors related to the individual* was highlighted as being important for participant adherence to CBGEP. Aspects such as participant characteristics of commitment, perseverance, preference for a routine or structure in their week, a desire to stay active in order to maintain independence, and being physically active in the past have been similarly reported by Chiang et al. [47] in their study of the experiences of ethnic older people (mean age 76 years, 85% female) in CBGEP. What this current study adds is the way that in some situations, participant circumstances appeared to play a role in their ongoing adherence. For example, for participants who lived alone, the opportunity to leave the house and be with others was important. For others, the personal circumstances of being a carer for their spouse meant that the CBGEP served as an opportunity to have time to themselves. In this way there was a sense that the CBGEP acted as a mental escape. The CBGEP afforded participants the opportunity to have time away when they were not required to function in another role, for example in their caring role. Participants commented that it was a time when they could forget their day-to-day worries and switch off, perhaps escaping mentally. This concept of escapism has similarly been reported in relation to regular, long-term exercise adherence [48] and in relation to pleasure as a source of immersion through which the focus on the exercise facilitates an escape [49]. However, this notion of escape has not been noted as a factor in relation to older peoples CBGEP adherence before.

Personal motivations to continue adhering stemmed from participants desires to maintain health and independence. For some this was so they could remain strong and healthy enough to continue to provide practical support in their role of caring for their spouses or family. For others, the motivation was based on remaining well enough to continue to live independently in their own home. This motivation to remain independent has similarly been reported by Hartley and Yeowell [50] in their study of what would potentially influence older people’s adherence to CBGEP. This desire to keep healthy and maintain independence implied a view about caring for the future. This is a flourishing view of participants’ long-term health and wellbeing which could be inferred as a eudaimonic understanding of wellbeing [51]. There was also a sense that PA was something that their body needed and attending the programme was enjoyable and thus a pleasure. Therefore conceptually this could be suggested as a hedonic experience [51, 52]. The concepts of eudaimonia and hedonia are important because they are central to the study of wellbeing [52].

There was evidence from this study regarding the role of the *instructor* as an important construct for older people’s adherence to CBGEP. This is supported by the existing exercise literature around older people’s engagement in CBGEP where the instructor has been noted to play an important, but not isolated role [53].

Based on the findings of this current study it is suggested that *instructors* need to be knowledgeable and competent yet approachable and human. It is this notion of the way participants were treated in a humanised manner which is unique knowledge generated from this current study. Instructors aided participants in maintaining agency by equipping them with information so they could self-select their exercise intensity depending upon their ability. This supported an environment whereby participants could exercise without criticism or judgement. Instructors were sensitive to the insider experiences and challenges faced by participants; they were not treated like an object. The instructors cared for and supported their participants. The fact that participants were treated in a humanised way suggests that this is a factor in the success of good programme adherence rates in these three cases.

This study highlighted the importance of considering several practical and structured features with regards *programme design* to support ongoing participant adherence to CBGEP. Aspects of programme design such as of location, affordability, adaptability of the exercises, safety, music, and opportunities to socialise have previously been noted in the literature around older people’s exercise adherence [47, 54–57]. This current study adds the suggestion that these features of programme design align with Herzberg’s dual factor theory [58]. This theory has become acknowledged as one of the most widely used theories in understanding motivation and satisfaction [59].

Herzberg argued that employees’ needs could be categorised as either relating to satisfaction (motivators), or to dissatisfaction (hygiene factors). This is relevant because when satisfiers and hygiene factors are present work outcomes are improved with a reduced rate of absenteeism [58, 60]. Thus, there may be parallels to be found in hygiene factors and satisfiers relating to CBGEP adherence.

In the context of CBGEP, it is suggested that several extrinsic factors such as location, affordability, or having an individual and adaptable content may function as hygiene factors. This would mean that the extrinsic factors do not necessarily contribute to participant satisfaction, but if those extrinsic factors were not present, they may lead to participant dissatisfaction with the programme, and potentially non-adherence. Conversely, it is proposed that satisfiers or motivators such as social factors or humanising elements contribute little to participant dissatisfaction but much to satisfaction and thus ongoing adherence. What this would mean in practice is that whilst factors such as location, affordability, music, and an adaptable content need to be present, these factors are not necessarily key in promoting participant satisfaction. However, they need to be present to prevent participant dissatisfaction. Thus, for this reason these programme design features do have a role to play in supporting ongoing participant adherence. Other elements reported in this study may serve to work more explicitly as motivators for example the social factors.

The roles of social interactions in CBGEP have been noted to add to participants’ networks and serve as a source of support, enjoyment and belonging [9, 50, 53]. This study corroborated these findings indicating that there was strong support for the social features of CBGEP in aiding participant adherence. The social, group dynamic of the programmes added to participants’ wellbeing by providing social, supportive environments which offered a means for older people to maintain social connectivity over the latter part of their lives. Maintaining these connections is important because there are increased health risks related to social isolation [23].

In particular, what this current study adds to the literature around the social aspects of CBGEP is further support for the importance of the sense of togetherness and belonging experienced by participants. This appeared to affect the way the participants felt about the group. It reveals the importance of the lifeworld as a viewpoint in understanding “an experienced world of meaning” [61] (p 55) with regards to CBGEP adherence. The notion of the lifeworld has been used as a philosophical foundation underpinning our perspectives for humanising healthcare [61]. These current authors would suggest that the lifeworld as an experienced world of meaning can also support perspectives for humanising older people’s adherence to CBGEP.

The physical health benefits of sustained exercise are well documented [62]. *Perceived physical gains* such as weight loss, cardiovascular fitness, and improved muscle strength were recognised by participants in this current study as an important outcome. However, the *perceived psycho-social gains* such as fun, enjoyment, and the notion of togetherness were also essential. Understanding these broader gains is important because emphasising the breadth of positive improvements associated with PA engagement is key in optimising uptake [63]. If CBGEP are to be promoted the psycho-social gains as well as the physical need to be affirmed. Participants seemed unlikely to adhere to a programme long-term that did not meet these psycho-social needs.

The CBGEP were noted to empower participants to self-manage painful long-term health conditions, such as back or neck pain. This is important not only in enabling individuals to maintain independence, but also in the context of financial challenges on the health service where self-management of long-term conditions may reduce health care costs [64]. Thus, CBGEP are of interest to those who commission services as an effective opportunity for supporting and empowering older people to manage their long-term health conditions.

The above findings are important because they add to the literature around understanding older people’s adherence to CBGEP. This is of relevance to practitioners and policy makers alike since CBGEP appear to be a possible means of encouraging sustained PA and social connectivity.

### Strengths and limitations of the study

#### Strengths

A strength of this study was the fact that the authors intentionally chose to study successful programmes (defined as adherence rates of ≥ 69.1% for ≥ 1 year), in a real-life context. To date, the authors are aware of only one study (from Canada) that chose participants in a real–life context who had displayed long-term adherence for the explicit purpose of studying ‘success’ [55]. Thus this current study is unique, being the first of its kind to study older people’s adherence to CBGEP in a real-life, UK context, from a long-term (≥ 1 year) perspective. The real-life context of the study is an important asset since participants recruited for an interventional randomised controlled trial are recruited differently to community exercisers, thus generalisability is limited between populations [65]. Findings from this current study are thus highly relevant in terms of real-life CBGEP. In addition, the cases included in this current study have all demonstrated ongoing sustainability to their programmes. The financial sustainability is particularly important in this present economic climate where programmes which have demonstrated sustainability are highly valued.

#### Limitations

This current study was subject to several limitations. This study only considered individuals who had been attending the CBGEP. There are many people who do not have this opportunity and the issue of how we engage with others who would benefit from this type of group was not addressed. Participants were self-selecting and thus might represent a highly motivated group of older people introducing potential bias. This means there is limited application to older people who drop out of CBGEP or to those who may be less social but still choose to exercise. Another limitation was the lack of data collected on ethnicity due to an omission by the researchers. Additionally, this study was limited to older people from one county in the South-West of England. Further cases should be selected from other counties using a similar study design to increase the applicability of the findings.

## Conclusion

The aim of this study was to understand how and why older people (≥ 60 years) have sustained long-term adherence The current study offers five unique insights into real-life programmes which have been successful in helping older people maintain adherence for a year or longer.

Firstly, there were *factors related to the individual* such as their desire to maintain their health and independence. Secondly, the *instructor* supported adherence through their personality, professionalism, and humanised approach. This helped participants feel cared for and established their sense of belonging to the group. Thirdly, several aspects of *programme design* such as location, affordability, the use of music, and adaptable exercise content promoted adherence. Fourthly, the *social*, group dynamic of the programme led to participants expressing a sense of togetherness and belonging which served as a conduit to adherence. Finally, *participants perceived physical and psycho-social benefits* such as weight loss, cardiovascular fitness, sense of fun, and enjoyment as contributing to their ongoing adherence.

An area for further research has been highlighted from this study based on the concept of escapism in supporting adherence to CBGEP for those who act as a main carer. This will add further understanding about how carers can be supported to continue in their vital roles whilst maintaining their own physical and mental well-being.

## Abbreviations

CBGEP: Community-based group exercise programme
FG: Focus group
NHS: National Health Service
NICE: National Institute for Health and Care Excellence
PA: Physical activity

## References

[1] National Institute for Health and Care Excellence (2013). Physical activity: brief advice for adults in primary care [online].

[2] Blair SN (2009). Physical inactivity: the biggest public health problem of the 21st century. British Journal Of Sports Medicine.

[3] Sun F, Norman IJ, While AE (2013). Physical activity in older people: a systematic review. BMC Public Health.

[4] Frew EJ, Bhatti M, Win K, Sitch A, Lyon A, Pallan M, Adab P (2014). Cost-effectiveness of a community-based physical activity programme for adults (Be Active) in the UK: an economic analysis within a natural experiment. Br J Sports Med.

[5] Löllgen H, Böckenhoff A, Knapp G (2009). Physical activity and all-cause mortality: an updated meta-analysis with different intensity categories. Int J Sports Med.

[6] Woodcock J, Franco OH, Orsini N, Roberts I (2011). Non-vigorous physical activity and all-cause mortality: systematic review and meta-analysis of cohort studies. International Journal Of Epidemiology.

[7] Dishman RK (1982). Compliance/adherence in health-related exercise. Health Psychol.

[8] Dishman RK (2001). The problem of exercise adherence: Fighting sloth in nations with market economies. Quest.

[9] Farrance C, Tsofliou F, Clark C (2016). Adherence to community-based group exercise interventions for older people: A mixed-methods systematic review. Prev Med.

[10] van Der Bij AK, Laurant MGH, Wensing M (2002). Effectiveness of physical activity interventions for older adults - A review. Am J Prev Med.

[11] Orsega-Smith E, Payne LL, Godbey G (2003). Physical and psychosocial characteristics of older adults who participate in a community-based exercise program. J Aging Phys Act.

[12] Ramsbottom R, Ambler A, Potter J, Jordan B, Nevill A, Williams C (2004). The effect of 6 months training on leg power, balance, and functional mobility of independently living adults over 70 years old. J Aging Phys Act.

[13] Belza B, Shumway-Cook A, Phelan EA, Williams B, LoGerfo JP, Snyder SJ (2006). The effects of a community-based exercise program on function and health in older adults: The Enhancefitness program. J Appl Gerontol.

[14] Hughes SL, Seymour RB, Campbell RT, Whitelaw N, Bazzarre T (2009). Best-practice physical activity programs for older adults: findings from the National Impact Study. Am J Public Health.

[15] Seguin RA, Heidkamp-Young E, Kuder J, Nelson ME (2012). Improved physical fitness among older female participants in a nationally disseminated, community-based exercise program. Health Educ. Behav..

[16] Hernandes NA, Probst VS, Da Silva RA, Januário RS, Pitta F, Teixeira DC (2013). Physical activity in daily life in physically independent elderly participating in community-based exercise program. Brazilian Journal of Physical Therapy.

[17] Wallace, R., Lees, C., Minou, M., Singleton, D. and Stratton, G., 2014. Effects of a 12-week community exercise programme on older people. Nursing Older People, 26, 20–26 10.7748/nop2014.02.26.1.20.e508 24471550

[18] Keogh JW, Rice J, Taylor D, Kilding A (2014). Objective benefits, participant perceptions and retention rates of a New Zealand community-based, older-adult exercise programme. J Prim Health Care.

[19] Munro JF, Nicholl JP, Brazier JE, Davey R, Cochrane T (2004). Cost effectiveness of a community-based exercise programme in over 65 year olds: Cluster randomised trial. J Epidemiol Community Health.

[20] King AC (2001). Interventions to promote physical activity by older adults. J. Gerontol. A Biol. Sci. Med. Sci..

[21] Bethancourt HJ, Rosenberg DE, Beatty T, Arterburn DE (2014). Barriers to and facilitators of physical activity program use among older adults. Clin. Med. Res..

[22] Steptoe A, Shankar A, Demakakos P, Wardle J (2013). Social isolation, loneliness, and all-cause mortality in older men and women. Proc Natl Acad Sci U S A.

[23] Holt-Lunstad J, Smith TB, Layton JB (2010). Social relationships and mortality risk: a meta-analytic review. PLoS Med.

[24] Reed SB, Crespo CJ, Harvey W, Andersen RE (2011). Social isolation and physical inactivity in older US adults: Results from the Third National Health and Nutrition Examination Survey. European Journal of Sport Science.

[25] Yin RK (2013). Case study research: Design and methods.

[26] Cohen L, Manion L, Morrison K (2007). Research methods in education [electronic resource].

[27] Merriam SB (2009). Qualitative research: a guide to design and implementation.

[28] Belza B, Topolski T, Kinne S, Patrick DL, Ramsey SD (2002). Does adherence make a difference? Results from a community-based aquatic exercise program. Nurs Res.

[29] National Institute for Health and Clinical Excellence. 2014. Behaviour change: Individual approaches [online]. NICE Public Health Guidance 49. Available from: https://www.nice.org.uk/guidance/ph49 . Accessed 10 Apr 2014

[30] Office for National Statistics. 2016. Neighbourhood statistics [online]. Office for National Statistics. Available from: http://www.neighbourhood.statistics.gov.uk/dissemination/. Accessed 30 Oct 2014

[31] Eisenhardt KM, Graebner ME (2007). Theory building from cases: Opportunities and challenges. Acad Manage J.

[32] Gill DP, Jones GR, Zou GY, Speechley M (2008). The Phone-FITT: a brief physical activity interview for older adults. J. Aging Phys. Act..

[33] Schröder H, Fitó M, Estruch R, Martínez-González MA, Corella D, Salas-Salvadó J, Lamuela-Raventós R, Ros E, Salaverría I, Fiol M, Lapetra J, Vinyoles E, Gómez-Gracia E, Lahoz C, Serra-Majem L, Pintó X, Ruiz-Gutierrez V, Covas MI (2011). A short screener is valid for assessing Mediterranean diet adherence among older Spanish men and women. Journal of Nutrition.

[34] Lubben J, Blozik E, Gillmann G, Iliffe S, Beck JC, Stuck AE, von Renteln Kruse W, Beck JC, Stuck AE (2006). Performance of an abbreviated version of the Lubben Social Network Scale among three European community-dwelling older adult populations. Gerontologist.

[35] Zimet GD, Dahlem NW, Zimet SG, Farley GK (1988). The Multidimensional Scale of Perceived Social Support. J Pers Assess.

[36] Hawley-Hague H, Horne M, Skelton DA, Todd C (2016). Review of how we should define (and measure) adherence in studies examining older adults’ participation in exercise classes. BMJ Open.

[37] Mack N, Woodsong C, MacQueen K, Guest G, Namey E (2005). Qualitative research methods: A data collector’s field guide.

[38] Pope C, Mays N (1995). Reaching the parts other methods cannot reach: an introduction to qualitative methods in health and health services research. BMJ.

[39] Holloway I, Wheeler S (2010). Qualitative research in nursing and healthcare.

[40] Krueger RA, Casey MA (2009). Focus Groups: A practical guide for applied research.

[41] NVivo qualitative data analysis software; QSR International Pty Ltd. Version 10, 2012.

[42] Gillham WEC (2000). Case study research methods.

[43] Braun V, Clarke V (2006). Using thematic analysis in psychology. Qual. Res. Psychol..

[44] Barbour RS (2001). Checklists for improving rigour in qualitative research: a case of the tail wagging the dog?. BMJ.

[45] Foster I, Jefferies J, Foster L (2014). Beginning statistics: An introduction for social scientists.

[46] Office for National Statistics. 2013. People, population and community [online]. Office for National Statistics. Available from: http://www.ons.gov.uk/peoplepopulationandcommunity/.Accessed 22 Apr 2016

[47] Chiang KC, Seman L, Belza B, Tsai HCJ (2008). “It is our exercise family”: experiences of ethnic older adults in a group-based exercise program. Prev Chronic Dis.

[48] Crossley N (2006). In the gym: Motives, meaning and moral careers. Body and Society.

[49] Phoenix C, Orr N (2014). Pleasure: A forgotten dimension of physical activity in older age. Soc Sci Med.

[50] Hartley SE, Yeowell G (2015). Older adults’ perceptions of adherence to community physical activity groups. Ageing & Society.

[51] Ryan RM, Deci EL (2001). On happiness and human potentials: A review of research on hedonic and eudaimonic well-being. Annu Rev Psychol.

[52] Huta V, Waterman A (2014). Eudaimonia and its distinction from hedonia: Developing a classification and terminology for understanding conceptual and operational definitions. J. Happiness Stud..

[53] Hawley-Hague H, Horne M, Skelton DA, Todd C (2016). Older adults’ uptake and adherence to exercise classes: Instructors’ perspectives. J. Aging Phys. Act..

[54] Fox K, Stathi A, McKenna J, Davis M (2007). Physical activity and mental well-being in older people participating in the Better Ageing Project. Eur J Appl Physiol.

[55] Dunlop WL, Beauchamp MR (2013). Birds of a feather stay active together: A case study of an all-male older adult exercise program. J Aging Phys Act.

[56] Garmendia M, Dangour A, Albala C, Eguiguren P, Allen E, Uauy R (2013). Adherence to a physical activity intervention among older adults in a post-transitional middle income country: A quantitative and qualitative analysis. J. Nutr. Health Aging.

[57] Kirby JB, Kluge MA (2013). Going for the gusto: Competing for the first time at age 65. J Aging Phys Act.

[58] Herzberg F (1987). One more time: How do you motivate employees?. Harv Bus Rev.

[59] DeShields OW, Kara A, Kaynak E (2005). Determinants of business student satisfaction and retention in higher education: Applying Herzberg’s two-factor theory. Int. J. Educ. Manag..

[60] Naumann E, Jackson D (1999). One more time: How do you satisfy customers?. Bus Horiz.

[61] Todres L, Galvin KT, Dahlberg K (2007). Lifeworld-led healthcare: Revisiting a humanising philosophy that integrates emerging trends. Med Health Care Philos.

[62] Chodzko-Zajko WJ, Proctor DN, Fiatarone Singh MA, Minson CT, Nigg CR, Salem GJ, Skinner JS (2009). Exercise and physical activity for older adults. Med Sci Sports Exerc.

[63] Brawley LR, Latimer AE (2007). Physical activity guides for Canadians: messaging strategies, realistic expectations for change, and evaluation. Can J Public Health.

[64] Naylor C, Imison C, Addicott R, Buck D, Goodwin N, Harrison T, Ross S, Sonola L, Tian Y, Curry N (2015). Transforming our health care system Ten priorities for commissioners [online]. The King’s Fund.

[65] Martin KA, Sinden AR (2001). Who will stay and who will go? A review of older adults’ adherence to randomized controlled trials of exercise. J. Aging Phys. Act..

